# Transcranial magnetic stimulation to understand pathophysiology and as potential treatment for neurodegenerative diseases

**DOI:** 10.1186/s40035-015-0045-x

**Published:** 2015-11-16

**Authors:** Zhen Ni, Robert Chen

**Affiliations:** Division of Neurology, Krembil Neuroscience Centre and Toronto Western Research Institute, University Health Network, University of Toronto, Toronto, ON Canada; 7MC-411, Toronto Western Hospital, 399 Bathurst Street, Toronto, ON M5T 2S8 Canada

**Keywords:** Alzheimer’s disease, Amyotrophic lateral sclerosis, Huntington’s disease, Parkinson’s disease, Transcranial magnetic stimulation

## Abstract

Common neurodegenerative diseases include Parkinson’s disease (PD), Alzheimer’s disease (AD), amyotrophic lateral sclerosis (ALS) and Huntington’s disease (HD). Transcranial magnetic stimulation (TMS) is a noninvasive and painless method to stimulate the human brain. Single- and paired-pulse TMS paradigms are powerful ways to study the pathophysiological mechanisms of neurodegenerative diseases. Motor evoked potential studied with single-pulse TMS is increased in PD, AD and ALS, but is decreased in HD. Changes in motor cortical excitability in neurodegenerative diseases may be related to functional deficits in cortical circuits or to compensatory mechanisms. Reduction or even absence of short interval intracortical inhibition induced by paired-pulse TMS is common in neurodegenerative diseases, suggesting that there are functional impairments of inhibitory cortical circuits. Decreased short latency afferent inhibition in AD, PD and HD may be related to the cortical cholinergic deficits in these conditions. Cortical plasticity tested by paired associative stimulation or theta burst stimulation is impaired in PD, AD and HD. Repetitive TMS (rTMS) refers to the application of trains of regularly repeating TMS pulses. High-frequency facilitatory rTMS may improve motor symptoms in PD patients whereas low-frequency inhibitory stimulation is a potential treatment for levodopa induced dyskinesia. rTMS delivered both to the left and right dorsolateral prefrontal cortex improves memory in AD patients. Supplementary motor cortical stimulation in low frequency may be useful for HD patients. However, the effects of treatment with multiple sessions of rTMS for neurodegenerative diseases need to be tested in large, sham-controlled studies in the future before they can be adopted for routine clinical practice.

## Background

Neurodegeneration involves progressive structural and functional loss of specific groups of neurons. The risk of being affected by a neurodegenerative disease increases dramatically with age. With increasing lifespan due to the population-wide health improvements, more individuals will be affected by neurodegenerative diseases in the coming decades. Common neurodegenerative diseases include Parkinson’s disease (PD) [1], Alzheimer’s disease (AD) [2], amyotrophic lateral sclerosis (ALS) [3] and Huntington’s disease (HD) [4]. The mechanisms underlying neurodegenerative diseases are multifactorial and include genetic and environmental factors. Current treatments for neurodegenerative diseases are symptomatic and there is no accepted disease modifying therapy to slow disease progression [1–4].

**Fig. 1:**
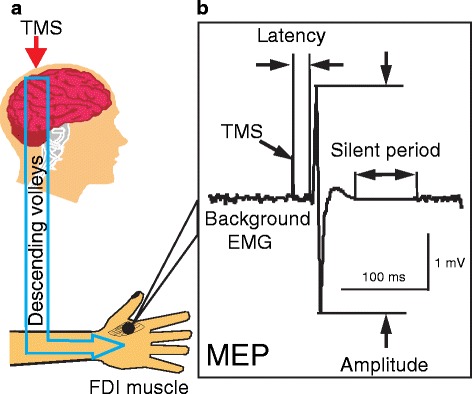
Transcranial magnetic stimulation and its measurements. **a** When TMS is applied to the primary motor cortex, it produces descending volleys in the spinal cord. This in turn activates the spinal motoneurons and a motor-evoked potential (MEP) can be recorded in the target muscle (e.g. FDI muscle) with surface EMG. **b** MEP measurements. When TMS is delivered during voluntary muscle contraction, an MEP is followed by a silent period with no background EMG activity. MEP latency is defined as the time from TMS delivery to the onset of MEP. MEP amplitude is usually measured as the peak-to-peak value. Silent period can be measured from the onset or the end of MEP to the first recovery of background EMG activity. EMG = electromyogram, FDI = first dorsal interosseous, MEP = motor evoked potential, TMS = transcranial magnetic stimulation. Modified from Ni et al., Transcranial magnetic stimulation in different current directions activates separate cortical circuits, Journal of Neurophysiology 2011, 105:749-756 [8]

Transcranial magnetic stimulation (TMS) is a noninvasive and painless method to stimulate the human brain [5, 6]. When stimulation is applied to the primary motor cortex (M1), it activates the corticospinal pathway and generates motor evoked potential (MEP) in the target muscles (Fig. 1) [6–8]. In addition to activation of corticospinal neurons, TMS also activates intracortical inhibitory and excitatory neural circuits in the M1. Repetitive TMS (rTMS) refers to application of trains of regularly repeating TMS pulses. These pulses temporally summate to cause changes in neural activity that can outlast the stimulation by minutes to hours [9]. Repeated applications of rTMS can produce even longer effects that last for weeks to months [7, 10]. Therefore, rTMS may be developed as a therapeutic tool for neurodegenerative diseases [7, 10]. In this article, studies investigating the pathophysiology and focusing on the development of treatments in PD, AD, ALS and HD will be reviewed.

## Parkinson’s disease

The motor symptoms of PD largely result from the degeneration of dopaminergic neurons in substantia nigra pars compacta. PD is associated with functional deficits in multiple brain areas, including basal ganglia nuclei, cerebellum and cortical areas [1]. We discuss here these functional deficits as tested by TMS measurements using several experimental designs. The main findings are listed in Table 1.

**Table 1:** Abnormalities in TMS measurements in neurodegenerative diseases^a^

Measurements		PD		AD	ALS	HD
		OFF	ON			
Single-pulse	MEP threshold	○	○	-	-/+^b^	+
	MEP amplitude	+	+	+	+	-
	Silent period	-	○/+	-	-	-
Paired-pulse	SICI	-	-/○	-	-	-
	ICF	○	○	○	+	×
	LICI	-	-	×	×	×
	SAI	○	-	-	×	-
	LAI	-	-	×	×	×
	IHI	−^c^	×	-	-	×
Cortical plasticity	LTP-like effect^d^	−^e^	○^e^	-	×	-
	LTD-like effect^d^	×	×	-/○	×	-

*Abbreviations: AD* Alzheimer’s disease, *ALS* amyotrophic lateral sclerosis, *HD* Huntington’s disease, *PD* Parkinson’s disease, *OFF* off dopaminergic medication, *ON* on dopaminergic medication

+ increase; - decrease; × not tested; ○ normal

*ICF* intracortical facilitation, *IHI* interhemispheric inhibition, *LAI* long latency afferent inhibition, *LICI* long interval intracortical inhibition, *LTD* long-term depression, *LTP* long-term potentiation, *MEP* motor evoked potential, *SAI* short latency afferent inhibition, *SICI* short interval intracortical inhibition

Notes:

^a^TMS measurements with different stimulus parameters may lead to different results in testing cortical circuits in neurodegenerative diseases. We only list the most consistent findings in the literatures. Detailed discussion is in the main text of the review

^b^MEP threshold increased in ALS but could be decreased at early stage of the disease

^c^Only long latency IHI was decreased in PD patients with mirror movement. Such abnormality was found from both the less affected to more affected side and from the more affected to less affected side. Short latency IHI does not change

^d^LTP-like effects are tested by facilitatory repetitive stimulation protocols including high-frequency repetitive transcranial magnetic stimulation, intermittent theta burst stimulation and paired associative stimulation while LTD-like effects are tested by inhibitory repetitive stimulation protocols including low-frequency repetitive transcranial magnetic stimulation and continuous theta burst stimulation

^e^LTP-like cortical plasticity tested with paired associative stimulation is impaired in PD patients off medication. Dopaminergic medications restore the plasticity in non-dyskinetic patients but not in the dyskinetic patients

### Single-pulse TMS measurements for Parkinson’s disease

#### Motor threshold

Motor threshold is an important parameter of motor cortical excitability. Rest and active motor thresholds are defined as the minimum TMS intensities that elicit small but reproducible MEPs at rest and during voluntary muscle contraction, respectively [6]. The motor threshold reflects the excitability of the most sensitive group of neurons in the stimulated area in M1. Most studies have reported that rest motor threshold is normal in PD [11–13]. Involuntary contraction caused by tremor and rigidity may affect the measurement in PD. Active motor threshold in PD appears to be normal although a correlation between the degree of bradykinesia and active threshold has been reported [14]. In addition, MEP threshold does not change with medication status [11–13] or deep brain stimulation of the internal globus pallidus [15] or the subthalamic nucleus [16].

#### MEP amplitude

MEP amplitude (Fig. 1b) reflects the global excitability of cortical interneurons, corticospinal neurons and spinal motoneurons [7]. Increased MEP amplitude at rest in PD patients has been reported [17, 18]. Increased MEP amplitude in PD may be related to an imbalance towards disinhibition in the motor pathway. Studies that showed decreased cortical inhibition, increased cortical facilitation and changes in cortical plasticity in PD are discussed below. Patients with internal globus pallidus deep brain stimulation also showed larger MEP amplitude than controls regardless of whether the stimulation was turned on or off [15].

#### Silent period

When TMS is applied during voluntary contraction, a disruption of the ongoing muscle activity known as the silent period can be recorded following the MEP (Fig. 1b). The first part of the silent period is partly due to decreased spinal excitability. The latter part of the silent period mainly involves inhibitory effects at the cortical level, mediated by gamma-aminobutyric acid type B (GABA_B_) receptors [6, 19]. Shortening of the silent period in PD has been reported in many studies [20]. However, such abnormality may not be pronounced at low stimulus intensities [12]. Dopaminergic medication normalizes the shortened silent period in PD [12]. High doses of levodopa may even lengthen the duration beyond the normal range [15].

#### Tremor reset

An asymmetric 4-6 Hz resting tremor is a cardinal symptom of PD. Many PD patients also have postural tremor [1]. When stimulation is applied to the motor pathway, the tremor may be transiently disrupted. The reoccurrence of the tremor is then time-locked to the stimulation and this phenomenon is referred to as tremor reset. Mechanical perturbation which modulates spinal reflex pathways has very little effect on postural tremor in PD, suggesting that spinal circuits may not be involved in generating PD postural tremor [21]. TMS applied to M1 completely resets postural tremor in PD [22]. PD rest tremor can also be reset by M1 TMS, suggesting that the M1 is involved in both resting and postural tremor in PD. In addition, cerebellar TMS is effective in resetting the PD postural tremor but not rest tremor, suggesting that the cerebellum is involved in the generation or transmission of postural tremor but not rest tremor in PD [23].

### Intracortical circuits in Parkinson’s disease

The excitability of intracortical circuits in M1 can be investigated by a paired-pulse TMS paradigm. The effect of the first conditioning stimulus on the MEP elicited by the second test stimulus depends on the stimulus intensities, the interstimulus interval and the location of conditioning stimulus.

#### Short and long interval intracortical inhibitions

**Fig. 2:**
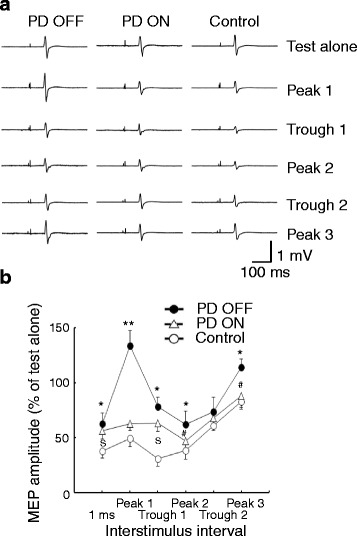
Abnormal SICI in PD patients. Example of recordings from representative subjects are shown in **a**. The top row represents the recordings with test stimulus alone and other five rows are recordings for paired-pulse stimulation at different interstimulus intervals. SICI was tested at the interstimulus intervals where short interval intracortical facilitation was at its peaks and troughs. An additional interval of 1 ms was also tested. Note that SICI was decreased at facilitatory peaks and troughs in the PD OFF medication state, and this was normalized in the PD ON state. The group data analysis is shown in **b**. The abscissa indicates the interstimulus interval. The ordinate indicates the degree of SICI. It represents the amplitude of paired-pulse induced MEP expressed as a percentage of the MEP amplitude induced by test stimulus alone. Values more than 100 % indicate facilitation and those less than 100 % indicate inhibition. Filled circles indicate MEP in PD patients OFF medication. Triangles indicate MEP in PD patients ON medication. Open circles indicate MEP in healthy controls. * *p* < 0.05, ** *p* < 0.01, comparing PD OFF to control. # *p* < 0.05, comparing PD OFF to PD ON. “S” *p* < 0.05, comparing PD ON to control. SICI was reduced in PD OFF compared to controls at an ISI of 1 ms, at short interval intracortical facilitation peak 1, trough 1, peak 2 and peak 3. Reduced SICI in PD OFF compared to PD ON group was only found at facilitatory peaks. SICI for PD ON was still decreased compared to controls at ISI of 1 ms and at facilitatory trough 1. MEP = motor evoked potential, PD = Parkinson’s disease, SICI = short interval intracortical inhibition. Modified from Ni et al., Increased motor cortical facilitation and decreased inhibition in Parkinson disease, Neurology 2013, 80:1746-1753 [11]. Promotional and commercial use of the material in print, digital or mobile device format is prohibited without the permission from the publisher Wolters Kluwer Health. Please contact healthpermissions@wolterskluwer.com for further information

Short interval intracortical inhibition (SICI) (Fig. 2) and intracortical facilitation can be tested with both conditioning and test stimuli delivered to the M1, with a subthreshold conditioning stimulus followed by a suprathreshold test stimulus. The test MEP is inhibited at interstimulus interval of 1-5 ms, and facilitated at interval of 7-30 ms [24]. SICI is enhanced by positive allosteric modulators of GABA_A_ receptors, suggesting that SICI is likely mediated by GABA_A_ receptors [25–27]. The mechanism mediating intracortical facilitation remains unclear but activation of cortical glutamate circuits may be involved [6]. One early study showed that SICI was reduced in PD patients and levodopa partly normalized this impaired inhibition [12]. Subthalamic nucleus deep brain stimulation increased the reduced SICI both in the on and off medication states [16] while internal globus pallidus stimulation had little effect on SICI [15]. Later studies reported controversial results that SICI was normal in PD patients either on or off medication [28] and decreased SICI was found only at high conditioning intensities [13]. Interestingly, a recent study showed that short interval intracortical facilitation, which is caused by summation of activation of different facilitatory interneurons in the M1, is increased in PD patients [11]. Since the stimulus parameters (interstimulus interval and stimulus intensities) for SICI and short interval intracortical facilitation overlap considerably, decreased SICI (Fig. 2) may partly be explained by increased facilitation in PD [11]. Specifically, short interval intracortical facilitation at the first peak increased from about 200 % of test alone (MEP induced by test stimulus alone) in healthy controls to about 300 % of test alone in PD patients. Concurrently, SICI at the same interstimulus interval turned from inhibition (about 50 % of test alone) to facilitation (about 130 % of test alone). In addition, SICI was reported to be normal on the less affected side and be reduced on the more affected side in newly diagnosed PD patients [29]. The abnormal SICI with asymmetry was observed up to 1 year after diagnosis [30].

Long interval intracortical inhibition is elicited when a suprathreshold conditioning stimulus is applied 50-200 ms prior to the test stimulus and is likely mediated by GABA_B_ receptors [6]. Long interval intracortical inhibition is reported to be decreased in PD [28]. This is consistent with shortened silent period (related to GABA_B_ receptors) in PD. Using a triple-pulse TMS paradigm, it has been found that SICI is suppressed in the presence of long interval intracortical inhibition in a manner consistent with reduction in GABA release caused by presynaptic GABA_B_ inhibition. The suppressive effect of long interval intracortical inhibition on SICI seen in healthy controls is absent in PD patients. Dopaminergic medications do not normalize this deficit, suggesting that presynpatic inhibition is impaired in PD and the impairment may be a non-dopaminergic feature of PD [28].

#### Interhemispheric inhibition

Interhemispheric inhibition can be measured by two TMS coils placed on bilateral M1s. Both conditioning and test stimuli are suprathreshold. Short and long latency interhemispheric inhibitions peak at interstimulu intervals of ~10 and ~50 ms. Inhibition is likely produced by interhemispheric inputs largely mediated through the corpus callosum [31]. There is less long latency interhemispheric inhibition in PD patients with mirror movement than those without mirror movement, suggesting that deficits in transcallosal function may contribute to mirror activity in PD. Such abnormality is found for long latency interhemispheric inhibition from both the less affected to more affected side and from the more affected to less affected side. There is no significant abnormality in short latency interhemispheric inhibition in PD [32].

#### Afferent inhibition

**Fig. 3:**
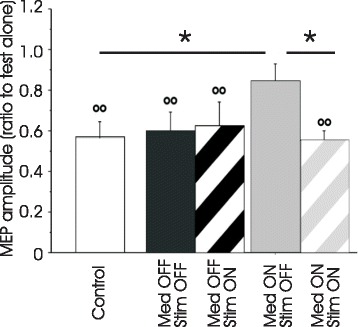
Short latency afferent inhibition in Parkinson’s diseasepatients with subthalamic nucleus deep brain stimulation. The abscissa indicates the different experimental conditions. The ordinate indicates the degree of short latency afferent inhibition. It represents the amplitude of paired-pulse induced MEP expressed as a ratio of the MEP amplitude induced by test alone. Values more than 1 indicate facilitation and those less than 1 indicate inhibition. * *p* < 0.05, comparing patients at ON medication OFF stimulation state to healthy controls and patients at ON medication ON stimulation state. The ring asterisks above the columns represent significant inhibition compared to test alone. Note that short latency afferent inhibition was normal in Parkinson’s disease patients at OFF medication state while it was reduced at ON medication state. Reduced inhibition at the ON medication state was normalized by the deep brain stimulation. MEP = motor evoked potential. Modified from Sailer et al., Subthalamic nucleus stimulation modulates afferent inhibition in Parkinson disease, Neurology 2007, 68:356-363 [34]. Promotional and commercial use of the material in print, digital or mobile device format is prohibited without the permission from the publisher Wolters Kluwer Health. Please contact healthpermissions@wolterskluwer.com for further information

Afferent input activated by electrical peripheral nerve stimulation inhibits the contralateral M1. Short (SAI) and long latency afferent inhibition refer to the inhibitory phases at interstimulus intervals of ~20 and ~200 ms. Cholinergic and GABA mediated pathways are involved in generating SAI, whereas transmitter involved in long latency afferent inhibition is not known [6, 8]. Figure 3 showed that SAI inhibited the MEP induced by test stimulus to about 60 % of its initial size. SAI is normal in PD off dopaminergic medications, but is reduced on medication state (MEP conditioned by electrical peripheral nerve stimulation was about 80 % of test alone). SAI probably represents a direct interaction between the sensory inputs and the M1. This pathway is unaffected by PD but is altered by dopaminergic medication and may contribute to the side effects of dopaminergic drugs. Long latency afferent inhibition is reduced in PD patients independent of their medication status, and probably involves indirect interactions between sensory inputs and the M1 via the basal ganglia or other cortical areas. This defective sensorimotor integration may be a non-dopaminergic manifestation of PD [33]. In addition, reduced SAI in the on medication state could be restored by subthalamic nucleus deep brain stimulation (Fig.3) and reduced long latency inhibition was partially normalized by the subthalamic stimulation in the on medication state [34]. Furthermore, such normalization of SAI and long latency afferent inhibition with subthalamic nucleus deep brain stimulation only occurred at 6 months but not at 1 month after implantation of stimulation electrodes and these effects were accompanied by normalization of proprioception (spatial and distance errors) [35]. Normalization of afferent inhibition with delayed time course suggests that the effect of subthalamic nucleus deep brain stimulation is related to the plastic changes in basal ganglia and cortical circuits produced by the chronic stimulation. In addition, the modulation of intracortical circuits by afferent inputs can be tested with a triple-pulse TMS paradigm. While long interval intracortical inhibition is reduced by long latency afferent inhibition in healthy controls, such modulation of long interval intracortical inhibition by afferent inputs is impaired in PD patients in both off and on medication states, which is manifested as similar degree of long interval intracortical inhibition in the presence of long latency afferent inhibition compared to that without afferent inhibition [33].

#### Cerebellar inhibition

Cerebellar inhibition refers to the phenomenon that stimulation over the cerebellum suppresses the MEP produced by contralateral M1 TMS delivered 5 to 7 ms later. Cerebellar inhibition is mediated by the cerebellothalamocortical pathway. Cerebellar TMS activates cerebellar Purkinje’s cells that inhibit the deep cerebellar nuclei, which has an excitatory projection to the motor cortex via the ventral thalamus [6]. Cerebellar inhibition is decreased in PD. Decreased inhibition correlated with the degree of reset of postural tremor caused by cerebellar stimulation, suggesting that the deficits on the cerebellothalamocortical pathway may be related to the tremor generation in PD [23].

#### Connectivity between the basal ganglia and M1

Inputs from the basal ganglia modulate M1 excitability. In PD patients with subthalamic nucleus deep brain stimulation, subthalamic stimulation leads to cortical evoked potential on the scalp with peak latencies of ~3 and ~20 ms [36]. Moreover, single pulse subthalamic stimulation produced two phases of MEP facilitation at 2-4 ms and 21-24 ms after the stimulation. The time course of MEP facilitation coincides with that of the evoked potentials recorded at the scalp. Antidromic conduction along the corticosubthalamic pathway likely mediates the early phase of facilitation while the late phase is likely mediated by synaptic transmission through the basal ganglia-thalamo-cortical circuit [36].

### Cortical plasticity in Parkinson’s disease

**Fig. 4:**
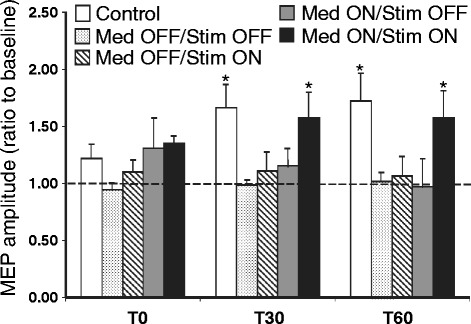
Motor cortical plasticity induced by paired associative stimulation in Parkinson’s disease with subthalamic nucleus deep brain stimulation. The abscissa indicates the time points (0, 30 and 60 min) after the intervention of paired associative stimulation. The ordinate indicates the MEP amplitude after the intervention. The values are expressed as a ratio to the MEP amplitude at baseline (before intervention). Values more than 1 indicate facilitation and those less than 1 indicate inhibition. White columns represent healthy controls. Columns with dots represent patients at medication OFF and deep brain stimulation OFF state. Hatched columns represent patients at medication OFF and stimulation ON state. Grey columns represent patients at medication ON and stimulation OFF state. Black columns represent patients at both medication and stimulation ON state. Note that cortical plasticity was impaired in the patients compared to healthy controls. Impaired cortical plasticity was only restored at the medication ON and deep brain stimulation ON state. * *p* < 0.05, comparing MEP at different time points to that at baseline (before intervention). MEP = motor evoked potential. Modified from Kim et al., Effects of subthalamic nucleus stimulation on motor cortex plasticity in Parkinson disease, Neurology 2015, 85:425-32 [39]. Promotional and commercial use of the material in print, digital or mobile device format is prohibited without the permission from the publisher Wolters Kluwer Health. Please contact healthpermissions@wolterskluwer.com for further information

Cortical plasticity can be tested by paired associative stimulation, which involves repetitive application of electrical peripheral nerve stimulation followed by TMS to M1. If peripheral stimulation precedes TMS by ~25 ms, the two stimuli arrive at the M1 at about the same time and lead to MEP facilitation in M1 [37]. This type of cortical plasticity is impaired in PD patients off medication. Dopaminergic medications restore the plasticity induced by paired associative stimulation in non-dyskinetic PD patients but not in the dyskinetic PD patients, suggesting that the development of dyskinesia is associated with greater disturbance of cortical plasticity [38]. In more advanced PD patients implanted with subthalamic nucleus deep brain stimulation, restoration of plasticity with paired associative stimulation was only observed in the medication on and stimulation on state (Fig. 4) [39]. Specifically, MEP amplitude 30 and 60 min after the paired associative stimulation increased to about 150 % of that at baseline in healthy controls. In patients with either medication off or deep brain stimulation off, MEP amplitude after paired associative stimulation was still about 100 % of baseline. When the patients were at both stimulation and deep brain stimulation on state, MEP after paired associative stimulation was facilitated to the similar level to that in healthy controls (about 150 % of baseline). The result suggests that the restoration of cortical plasticity is related to the clinical benefits of deep brain stimulation in PD. On the other hand, MEP facilitation induced by paired associative stimulation on the less affected side in the newly diagnosed PD patients was increased while the same protocol did not produce MEP facilitation on the more affected side in these patients [29]. Furthermore, the asymmetric responses to paired associative stimulation was found up to one year after diagnosis and the degree of asymmetry correlated with asymmetry in clinical rating scores for the less and more affected sides [30]. Intermittent theta burst stimulation produces MEP facilitation in healthy subjects [40]. Similar MEP facilitation has been reported in PD patients [41] whether in the medication on or off state [42]. However, this form of cortical plasticity may be impaired in more advanced PD patients [43].

### Therapeutic rTMS in Parkinson’s disease

rTMS involves trains of TMS pulses delivered with durations ranging from several seconds to several minutes at various frequencies and intensities. The effects of these pulses temporally summate to cause greater and longer duration of changes in neural activity than those from single-pulse TMS. Generally, high-frequency rTMS potentiates MEP and low-frequency rTMS suppresses MEP when delivered to the M1 [6, 7]. Since the effects of a single session of rTMS can last for several hours and repeated sessions may last for months, rTMS is a potential treatment for neurological disorders.

While many studies investigated the effects of rTMS on PD symptoms, the results were variable [44]. A large placebo effect with sham stimulation has been observed [45]. Meta-analyses found that high-frequency rTMS improved motor symptoms in PD patients while low-frequency rTMS had little benefit [44, 46] (Table 2). Intermittent theta burst stimulation has also been used to treat PD motor symptoms. However, a study that used eight sessions of stimulation over two weeks did not find long-term effect on PD motor symptoms but there were benefits on mood [41]. Low-frequency rTMS has been used to treat levodopa induced dyskinesia. One Hz rTMS over the M1 [47] with a two-week course produced short term improvement in levodopa induced dyskinesia [48]. Similar improvement was confirmed by a sham-controlled study. However, significant improvement in dyskinesia after rTMS was only found when compared to baseline and the difference between real and sham stimulations was not significant [49].

**Table 2:** Therapeutic repetitive TMS protocols for neurodegenerative diseases

	Protocol^a^	Target	Potential beneficial effects
Parkinson’s disease	Facilitatory	M1, SMA, PMd	Improve motor symptoms^b^, mood^c^
	Inhibitory	M1, cerebellum	Improve levodopa induced dyskinesia
Alzheimer’s disease	Facilitatory	DLPFC	Improve memory, cognition
Amyotrophic lateral sclerosis	Inhibitory	M1	Improve motor symptoms
Huntington’s disease	Inhibitory	SMA, M1	Improve chorea

*Abbreviations: DLPFC* dorsolateral prefrontal cortex, *M1* primary motor cortex, *PMd* dorsal premotor cortex, *SMA* supplementary motor area

Notes:

^a^Facilitatory protocols include high-frequency repetitive transcranial magnetic stimulation and intermittent theta burst stimulation; inhibitory protocols include low-frequency repetitive transcranial magnetic stimulation and continuous theta burst stimulation

^b^Facilitatory protocols with different stimulus parameters applied to M1, SMA and PMd may improve motor symptoms in PD

^c^A study of eight sessions of intermittent theta burst stimulation of M1 over two weeks reported benefits in mood in PD

Stimulation of other areas outside the M1 may also be effective. In particular, a sham-controlled study with a relatively large sample size reported that 5 Hz rTMS applied to the supplementary motor area significantly improved the clinic rating scores and bradykinesia in PD patients [50]. Continuous theta burst stimulation, a type of inhibitory rTMS, delivered to the cerebellum improved levodopa induced dyskinesia in PD [51]. In addition, 5 Hz rTMS over dorsal premotor cortex facilitated MEP in healthy controls but not in PD patients off medications. After levodopa administration, the facilitatory effect of premotor cortical stimulation on the motor cortex was restored [52].

## Alzheimer’s disease

AD is the most common form of dementia and is characterized by progressive neuronal degeneration. The degenerative process leads to atrophy initially in the hippocampus and entorhinal cortex, then progressively expanding into wide areas including the cerebral cortex and subcortical regions [2, 53]. Mild cognitive impairment (MCI) is considered a transitional stage between normal aging and clinically probable AD. The functional impairments in AD measured with TMS paradigms are summarized in Table 1.

### Single-pulse TMS measurements for Alzheimer’s disease

#### Motor threshold

Rest motor threshold is decreased in AD [54]. However, the threshold is preserved in patients with early disease [55] and in patients with MCI [56], suggesting that reduction in rest threshold may be a compensatory mechanism for the neuronal loss in motor cortical areas and may reflect a functional change in these areas with disease progression. Reduction in active motor threshold in AD has also been reported [54].

#### MEP amplitude and silent period

MEP amplitude may be normal at early stage of AD [54] but is increased in patients at advanced stages [57]. Interestingly, a TMS mapping study showed that the hotspot did not change while the center of gravity for MEP amplitude shifted in a fronto-medial direction in patients with mild to moderate AD, suggesting an early cortical reorganization in AD [58]. Silent period is shortened in moderate to severe AD, suggesting that AD may impair the function of GABA_B_ receptor mediated inhibitory circuits in M1 at late disease stages [59].

### Intracortical circuits in Alzheimer’s disease

#### Short latency afferent inhibition

Reduction in SAI is significant at many disease stages in AD [54, 56, 60–64] and this is consistent with postmortem studies showing central cholinergic impairment in AD [53]. Decreased SAI correlated with the degree of memory loss [60] and the degree of euphoric manic state in AD [61]. These correlations may be explained by the cholinergic dysfunction in temporo-limbic areas such as hippocampus, entorhinal cortex and amygdala. Administration of a single dose of rivastigmine (an acetylcholinesterase inhibitor) restored the decreased SAI in AD [54]. Since decreased SAI was found in early AD [62] and even in amnesic MCI patients [63, 64], it is a potential biomarker for the diagnosis of AD.

#### Other intracortical circuits

Reduction in SICI has been reported [65]. The degree of disinhibition correlated with the severity of AD [65]. However, other studies reported no difference in SICI between patients and controls [54, 57]. Although AD may be related to changes in cortical glutamatergic transmission [53, 58], intracortical facilitation in AD and MCI patients were normal [54, 57, 64]. Interhemispheric inhibition is decreased in amnesic MCI patients [64]. However, decreased inhibition does not correlate with the scores of mini-mental status examination or reduced SAI, suggesting that structural or functional impairment in transcallosal connection may occur earlier than the cognitive impairments in MCI [64].

### Cortical plasticity in Alzheimer’s disease

Long term potentiation-like cortical plasticity is impaired in AD. Five Hz rTMS which produced MEP increase in healthy controls decreased MEP in AD patients [66]. Similarly, paired associative stimulation [67] and intermittent theta burst stimulation [68], which induce MEP facilitation in normal subjects, also led to reduced cortical excitability in AD patients. Whether long term depression-like effect is altered in AD is controversial. One Hz rTMS, which produced MEP inhibition in healthy controls [47], had no effect in AD patients [69]. However, MEP inhibition with continuous theta burst stimulation in AD was normal [68].

### Therapeutic rTMS for Alzheimer’s disease

The assumption in AD that memory deficit is related to functional impairment in dorsolateral prefrontal cortex [70] makes this cortical area a common target of therapeutic intervention (Table 2). It was reported that application of 20 Hz rTMS to both the left and right dorsolateral prefrontal cortex improved the accuracy of an action naming task in both mild and moderate to severe AD patients [71]. A subsequent study with daily 20 Hz rTMS with 2000 pulses applied to the left dorsolateral prefrontal cortex for 2 or 4 weeks showed long-lasting improvement (8 weeks) in language comprehension in moderate AD patients [72]. Another study reported that 20 Hz right side followed by left side dorsal lateral prefrontal cortical stimulation applied for 5 days improved the score of mini-mental status examination in AD patients. On the other hand, 1 Hz stimulation applied in the same order (right followed by left side stimulation) had no effect, suggesting that facilitatory but not inhibitory stimulation has therapeutic effects in AD [73]. However, another study reported that a single session of inhibitory 1 Hz rTMS over right dorsolateral prefrontal cortex increased the recognition memory performance in both healthy controls and MCI patients [74].

## Amyotrophic lateral sclerosis

ALS is a rapidly progressive neurodegenerative disorder of the motoneurons in the M1, brainstem and spinal cord. A combination of upper and lower motoneuron dysfunction comprises the clinical ALS phenotypes [3].

### Single-pulse TMS measurements in amyotrophic lateral sclerosis

MEP threshold is increased in ALS [75, 76] (Table 1). However, a longitudinal study reported reduced MEP threshold at early stage of the disease, which may explain muscle fasciculation with motor neuronal changes at this stage [76]. Central motor conduction time is prolonged in ALS, reflecting axonal degeneration of the fast conducting fibers of corticospinal neurons [76]. MEP amplitude increases in sporadic [77] and familial forms of ALS [78], prominently in the early stage of the disease. In addition, MEP amplitude correlates with traditional measurement of peripheral nerve functions (compound muscle action potential) and with measurement for axonal excitability in ALS, suggesting an association between cortical hyperexcitability and motoneuron degeneration [77]. Reduction in duration of silent period is also prominent at early stage of ALS, indicating degeneration or dysfunction of inhibitory interneurons with reduced GABA_B_ receptor functions in ALS [77, 78].

### Intracortical circuits in amyotrophic lateral sclerosis

SICI is reduced or absent in ALS [77–79] (Table 1). This is consistent with the pathological finding of degeneration of inhibitory cortical interneurons in ALS [80]. In addition, reduction in SICI precedes the clinical development of familial ALS, which may help in establishing the diagnosis [78]. Intracortical facilitation is increased in ALS [77, 78], suggesting that glutamate mediated excitotoxicity may be involved in motoneuron hyperexcitability. Involvement of glutamate circuit in ALS pathophysiology is supported by the interesting finding that glutamate antagonist riluzole restored the decreased SICI in ALS patients [79]. Interhemispheric inhibition is also decreased in ALS [81]. Taken together, the reduction in cortical inhibition and increase in cortical facilitation may be related to hyperexcitability of cortical motoneurons in ALS patients.

### Cortical plasticity and therapeutic rTMS for amyotrophic lateral sclerosis

Two weeks of daily sessions of 5 Hz rTMS only had transit benefit on motor performance and the quality of life in ALS patients [82]. Twenty Hz rTMS even showed a tendency to accelerate disease progression [83]. These studies suggest that facilitatory rTMS may have minor beneficial effects or may be harmful in some circumstances in ALS. Inhibitory 1 Hz rTMS showed slight benefits in two ALS patients [83], supporting the idea that down regulation of hyperexcited motoneurons may improve symptoms (Table 2). Subsequent studies by the same group tested the effect of inhibitory rTMS with a design delivering 5 consecutive daily sessions of continuous theta burst stimulation per month. Long term benefit was observed in studies with different durations (0.5-2 years) and different sample sizes. A 26-month trial in a single case reported a slower rate of deterioration with stimulation compared to baseline. The strongest beneficial effect was found in the first 12 months with stimulation [84]. A six-month study reported a slight but significantly slower disease progression in 7 patients with real stimulation compared to 8 patients with sham stimulation [85]. Unfortunately, a one-year follow up double blinded placebo-controlled study with more patients failed to confirm the positive effects of the previous studies [86].

## Huntington’s disease

HD is a genetic neurodegenerative disease due to pathological expansion of the triplet cytosine-adenine-guanine (CAG) repeat in the *Huntingtin* gene in chromosome 4, which results in an excessively long polyglutamine stretch in protein *Huntingtin* and eventually causes loss of GABAergic neurons in striatum [4]. HD is characterized by a triad of symptoms with motor, cognitive and psychiatric disturbances.

### Single- and paired-pulse TMS measurements in Huntington’s disease

Higher rest and active motor thresholds and smaller rest MEP size compared to healthy controls were found in both very early symptomatic HD patients and HD gene carriers [87]. However, probably due to the small sample size and phenotypic heterogeneity, other studies found no difference in MEP threshold [88, 89] or amplitude [88] between HD patients and controls (Table 1). Although silent period may be normal at the early or preclinical stage of HD [87], progressive shortening in silent period with functional decline was found in symptomatic patients at two-year follow up [90]. The finding is consistent with HD pathology with GABAergic neuronal loss in the brain and suggests that the silent period may be a potential biomarker of the disease progression. Several studies reported normal SICI in symptomatic HD patients [89, 91]. However, the results may be confounded by inclusion of patients with chorea due to various etiologies. The conditioning stimulus intensity for producing same degree of SICI was found to be increased in early and even in the preclinical stage of the disease [87]. SAI was decreased in the same group of patients [87]. These studies with single- and paired-pulse measurements support the view that cortical functional impairments occur early in HD.

### Cortical plasticity and therapeutic rTMS in Huntington’s disease

Cortical plasticity is impaired in HD. MEP facilitation produced both by 5 Hz rTMS [92] and by paired associative stimulation [93] were reduced in HD patients. MEP inhibition produced by continuous theta burst stimulation was decreased in early symptomatic HD patients and HD gene carriers [88]. The use of rTMS as a treatment for HD has been studied (Table 2). One Hz but not 5 Hz rTMS applied to the supplementary motor area reduced chorea scores in HD patients, suggesting that suppression of supplementary motor cortical excitability may lead to improvement in HD symptoms [94]. Interestingly, dramatic improvement in dyskinesia lasting for 24 h after a single session of continuous theta burst stimulation to M1 was reported in a case of hemichorea secondary to midbrain and caudate hemorrhage [95].

## Conclusions and final remarks

Although aging is the greatest risk factor for neurodegenerative diseases, many neurodegenerative diseases can be caused by genetic mutations and are associated with protein misfolding and degradation. The effects of neurodegeneration can be found in many different levels of neuronal circuitry ranging from the molecular level to the systems level. Studies using animal models and neuroimaging techniques are searching for the biomarkers for neurodegenerative diseases. Development of disease modifying therapies such as gene therapy, stem cell transplant and neuroprotective agent are actively being pursued [96].

TMS provides a non-invasive and powerful process to investigate the synaptic activity and to manipulate the synaptic plasticity in human cortex at the systems level. Studies with single- and paired-pulse TMS showed abnormal cortical excitability in patients with neurodegenerative diseases. rTMS within established guidelines is safe for the patients with neurodegenerative diseases and showed symptomatic benefit in some studies. Several major issues should be considered for future studies that focus on better understanding of the pathophysiology and novel therapeutics for neurodegenerative diseases. First, the protocols with diagnostic or therapeutic potentials should be translated into clinically practical applications. Currently, this is largely limited by the fact that many TMS measurements have large within-subject and between-subject variations [6, 7]. Second, there is no current biomarker which can confirm the diagnosis of neurodegenerative disease at early stage and monitor the disease progression. Recently, genetic (such as genome sequencing, proteomics) and neuroimaging (such as positron emission tomography, functional magnetic resonance imaging) approaches are being undertaken to identify potential biomarkers for neurodegenerative diseases. Future studies combining TMS with these techniques may provide new opportunity to find clinically useful biomarkers for neurodegenerative diseases. Third, the current evidence showed that the beneficial effects of rTMS for neurodegenerative diseases are mild to moderate and short-lasting. While multiple sessions of rTMS may extend the clinical benefit, development of rTMS into a practical treatment requires large, sham-controlled studies and may need to introduce new stimulation parameters. In addition, the combination of rTMS with other traditional therapeutic methods such as medications and deep brain stimulation may lead to new treatment strategies for neurodegenerative diseases.

## Abbreviations

AD: Alzheimer’s disease
ALS: amyotrophic lateral sclerosis
GABA: gamma-aminobutyric acid
HD: Huntington’s disease
M1: primary motor cortex
MCI: mild cognitive impairment
MEP: motor evoked potential
PD: Parkinson’s disease
rTMS: repetitive transcranial magnetic stimulation
SAI: short latency afferent inhibition
SICI: short interval intracortical inhibition
TMS: transcranial magnetic stimulation
